# The Comparison of Inflammatory Cytokines (IL-6 and IL-18) and Immune Cells in Japanese Encephalitis Patients With Different Progression

**DOI:** 10.3389/fcimb.2022.826603

**Published:** 2022-04-07

**Authors:** Yun Zhou, Peiyu Bian, Hong Du, Tao Wang, Mengyuan Li, Haifeng Hu, Chuantao Ye, Xuyang Zheng, Ying Zhang, Yinfeng Lei, Zhansheng Jia, Jianqi Lian

**Affiliations:** ^1^Department of Infectious Diseases, Tangdu Hospital, Air Force Medical University, Xi’an, China; ^2^Department of Microbiology, School of Preclinical Medicine, Air Force Medical University, Xi’an, China; ^3^The Center of Infectious Diseases and Liver, Xi’an International Medical Center Hospital, Xi’an, China

**Keywords:** Japanese encephalitis virus, cytokines, CD4^+^T cells, B cells, prognosis

## Abstract

**Background:**

Japanese encephalitis virus (JEV) is the main cause of viral encephalitis in Asia. Nowadays, no effective and specific therapy for JE patients is available except supportive treatment. The fatality rate of JE patients is still about 30%, and more than half of survivors suffered from various neuropsychiatric sequelae. Thus, more attention should be paid to JE.

**Methods:**

In this study, a retrospective cohort of JE patients was collected and the general features of JE patients admitted into the Department of Infectious Diseases were analyzed. Meanwhile, the dynamic change of plasma cytokines and immune cells in JE patients with divergent prognosis was detected and analyzed.

**Results:**

We found a mounted proportion of adult/old patients in JE cases. The level of IL-6 and IL-18 increased in JE patients especially in fatal individuals. There was a continuous decreased percentage of CD4^+^ T and B cells in severe JE patients with fatal outcome compared with the surviving JE patients.

**Conclusions:**

The consistent high level of IL-6 and IL-18 in the plasma and low proportion of CD4^+^ T and B cells in the PBMCs might be the indicators of poor prognosis.

## Introduction

The incidence of JEV infection in children has decreased significantly since the launch of the nationwide JEV vaccination program in China in 2007 (Deng et al., 2020). However, the number of adult/old patients infected with JEV has mounted recently due to the change of socio-environmental factors (Zhang et al., 2018). Nowadays, there is no effective and specific therapy for JE patients except supportive treatment. Even with the advanced medical equipment, the fatality rate of JE patients is still about 30%, and more than half of the survivors developed various neuropsychiatric sequelae (Campbell et al., 2011). Thus, more attention should be paid to JEV-related research and treatment.

JEV belonging to the genus *Flavivirus* in the family Flaviviridae can induce critical arbovirus encephalitis which is the main cause of viral encephalitis in Asia. Through the bite of a JEV-infected mosquito, the virus can enter into the skin and infect the local Langerhans cells (LCs) and dendritic cells (DCs). JEV can proliferate in these infected cells at a low level and spread into the regional lymphoid node for further proliferation. Then, the virus disseminates into distant lymphoid nodes and finally into the central nervous system (CNS) through blood (Filgueira and Lannes, 2019). During early infection, the peripheral immune system can be activated and the induced inflammation contributes to the elimination of virus. Meanwhile, JEV can also inhibit the peripheral immune response and the escaping virus can enter into the CNS through the blood–brain barrier (BBB) (Mustafá et al., 2019). JEV as the neurotrophic virus can induce lethal neuroinflammation including the breakdown of BBB, death of neurons, activation of glia, and infiltration of immune cells into the CNS (Lannes et al., 2017).

Neurovirulence directly induced by JEV and immune-mediated damage are the main causes of neuro-impairment. Nowadays, neither specific initial signs of JEV infection nor demonstrated antiviral method to JEV treatment is available, leading to the failure of early block of virus (Turtle and Solomon, 2018). Meanwhile, the determinants of clinical progression and outcome of JE patients are not completely clear, which makes it vaguer for any clinical treatment plan. Proinflammatory cytokines, chemokines, and immune cells are all involved in JE progression. In JEV-infected mouse models, it has been established that B cell-mediated humoral immunity is vital for mice to fight against JEV infection (Wang et al., 2017). Meanwhile, the roles of TNF-α, IFN-γ, and IFN-α in JE progression are complicated (Lannes et al., 2017). Also, the effects of T cell- especially CD8^+^ T cell-mediated cellular immune, peripheral innate immune cells such as monocytes and NK on the progression of JEV-infected mice are also inconsistent among different labs (Sooryanarain et al., 2012; Turtle et al., 2016; Grifoni et al., 2020). According to previous clinical studies, the level of TNF-α, IL-6, IL-8, and RANTES in the cerebrospinal fluid (CSF) was elevated significantly in dead human JE cases than in survivors (Winter et al., 2004). However, there is no report on the dynamic level of cytokines and the proportion of peripheral immune cells in JE patients with different prognoses. Meanwhile, the correlation between these changes and the clinical outcome is elusive.

In JE patients, the peripheral and central immune inflammatory responses lead to clinical manifestations including fever, headache, convulsions, and consciousness impairment. The vital signs of severe patients are detected in the ICU, and the corresponding supportive treatments are given. However, some fatal cases eventually die after the extensive medical aid, leaving enormous financial burden and psychological distress to the families. Thus, besides research on effective intervention, exploring the signatures which correlate with the progression of grave cases is also necessary for the reasonable treatment. In this research, we detected the change of main cytokines and the types of immune cells from healthy individuals and JE patients with different gravity and outcomes aiming to find some cues that may indicate the progression of the cases.

## Materials and Methods

### Patients

There were about 149 patients diagnosed with JE (with anti-JEV IgM-positive and clinical JE characteristics) admitted into our department from January 2014 to October 2018. The gravity of JE patients was assessed by their doctors according to the clinical manifestations including temperature, neurologic symptoms, mental status, and consciousness. The mild patients were sent to common wards and the blood samples were collected immediately when they were hospitalized. The severe patients were admitted into intensive care units (ICU), and the blood sample collections were carried out continually with several days’ intervals until the end of the hospitalization. The course of the disease was calculated from the day of fever and headache onset. The control blood samples were obtained from healthy volunteers, who were negative of JEV and other diseases. The six healthy individuals were 3 male and 3 female; their average age was 36 (23-56). The research protocols were approved by the Scientific and Ethical Committee of Tangdu Hospital (TDLL-2018-03-267). All procedures were performed in accordance with the ethical standards laid down in the 1964 Declaration of Helsinki and its later amendments. The consent was obtained from each patient’s accompanying families.

### Materials

Antibodies anti-CD14-FITC, anti-CD16-APC, anti-CD56-PE, anti-CD3-APC, anti-CD4-FITC, anti-CD8-PE, and anti-CD19-PE were purchased from BD Biosciences (Heidelberg, Germany). The Luminex assay kit was purchased from Bio-Rad company (Hercules, CA, USA).

### Methods

#### Flow Cytometry

PBMCs were isolated using Ficoll–Hypaque density centrifugation (Sigma, St Louis, MO, USA). Cells were divided into three groups for staining respectively including CD19, CD3/CD4/CD8, and CD14/CD16/CD56 and then incubated on ice for 40 min. After being washed with PBS containing 1% FBS for three times, cells were detected using the flow cytometer.

#### Luminex Assay

The heparinized blood samples were centrifuged at 500 g for 10 min, and the plasma was collected and stored at -80°C. If not used immediately, PBMCs and plasma were cryopreserved in liquid nitrogen until used. The samples were thawed for the Luminex assay.

### Statistical Analysis

One-way ANOVA-LSD was applied to a three-group analysis. Mann–Whitney U test was used for two groups comparisons (SPSS software; version 24). GraphPad prism 6 was used for the figure processing.

## Results

### The General Information of JE Patients Admitted Into Tangdu Hospital During January 2014 to October 2018

**Figure 1:**
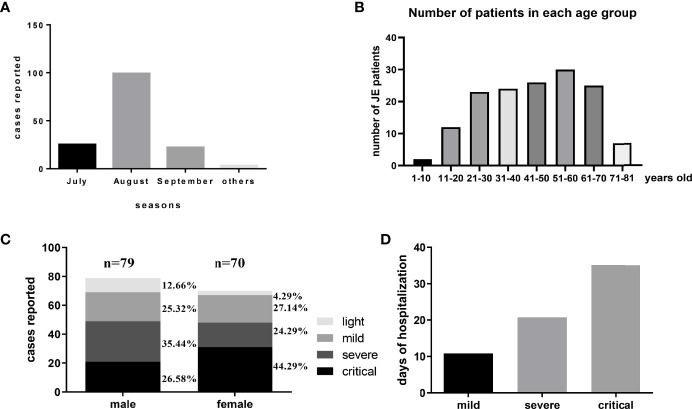
The general information of JE patients administered into Tangdu Hospital during January 2014 to October 2018. **(A)** There were about 149 patients diagnosed with JE administered into the Department of Infectious Diseases from January 2014 to October 2018. The distribution of JE cases was about July 25, August 99, September 22, and others 3. **(B)** The number of patients in each age group is about 1–10 years: 2, 11–20 years: 12, 21–30 years: 23, 31–40 years: 24, 41–50 years: 26, 51–60 years: 30, 61–70 years: 25, 71–81 years: 7. **(C)** There were 79 cases of male patients (light, mild, severe, critical) and 70 cases of female patients (light, mild, severe, critical). **(D)** The average days of hospitalization of each group were about mild 10.85 days, severe 20.76 days, and critical 35.12 days.

There were about 149 patients diagnosed with JE admitted into our department from January 2014 to October 2018. Among these JE sufferers, 120 patients survived while 29 patients (19.46%) failed to survive after supportive treatment. Compared with previously reported mortality of about 30%, the decreased mortality might be explained by the improved medical equipment provided by ICU. July, August, and September were still the main seasons of JE occurrence in accordance with the distribution of mosquitos (**Figure 1A**). Recent years, the increased incidence of adult JEV infection was more noticeable (Zhang et al., 2018). In our study, patients over 20 years old account for nearly 90% of the total JE patients (**Figure 1B**). According to our data, there were 79 male JE patients and 70 female patients and the proportion of fatal cases was a little higher in female compared to male (**Figure 1C**). There were no correlations between age and severity of JE-positive patients. Besides, there was no statistical significance among these four groups (see **Supplementary Material**). Obviously, the day of hospitalization was longer among the more serious JE patients (**Figure 1D**). Despite the significant decrease in JE incidence among children, the prevention to JEV infection cannot be ignored. The adult/old individuals especially those with cerebrovascular disease was vulnerable to JEV infection (Zhang et al., 2018). Most of the severe patients need to be supplied with oxygen, fluid, and corresponding treatment against high fever, coma, encephaledema, convulsions, and infection in ICU which needed enormous financial expenses. Some patients still failed to survive after a series of treatment and struggle. Meanwhile, the determinants for the severe patients to survive or not are unclear.

### Series of Cytokines in the Plasma Were Different Between the Healthyand JE Patients

**Figure 2:**
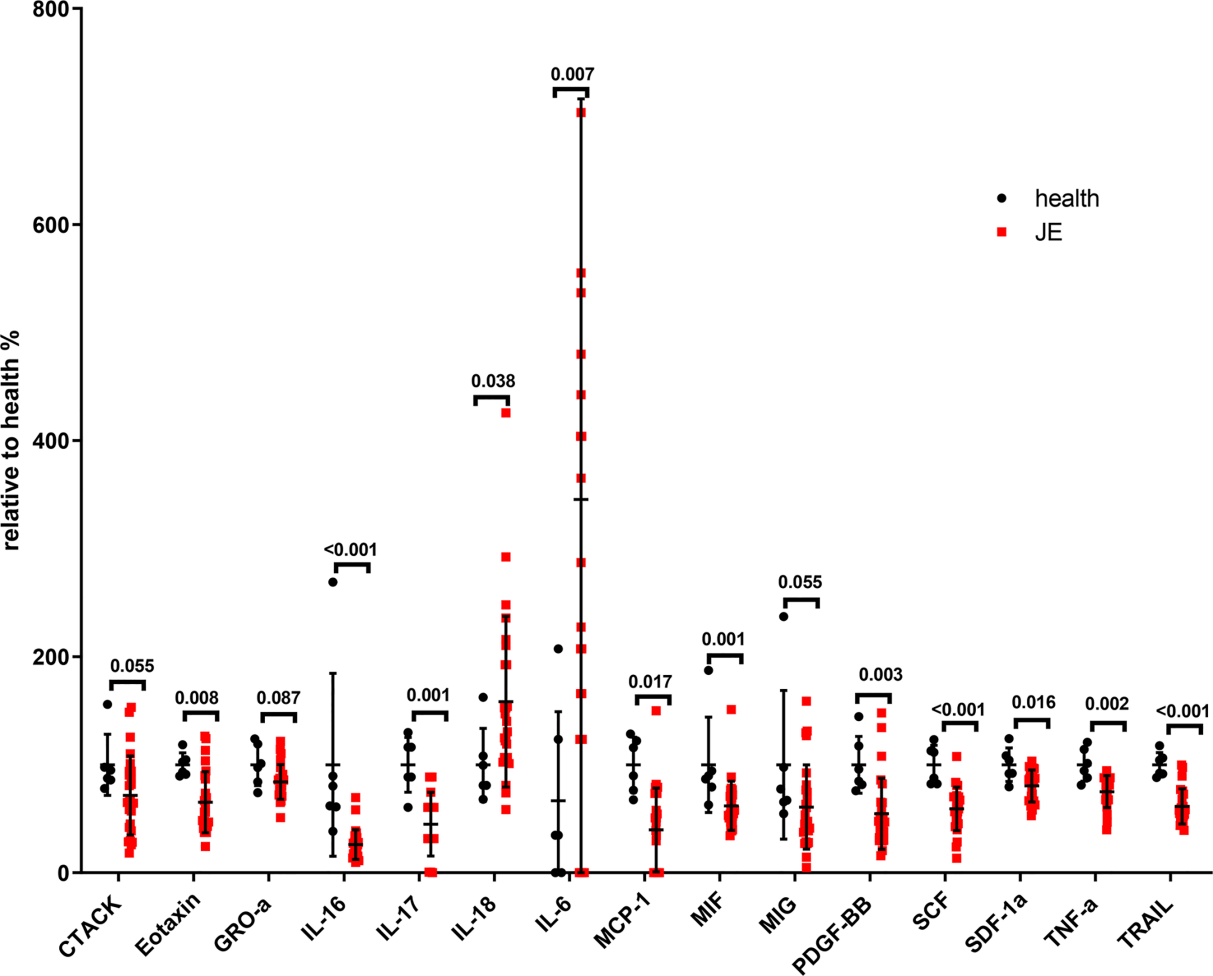
The significant change of total main cytokines in JE patients relative to the health. Series of cytokines in the plasm of healthy controls (n = 6) and JE patients (n = 26; these data were from the plasma of JE cases on the first day they were hospitalized. The Mann–Whitney U test was used to compare differences in median values between two groups. p <0.05 was considered statistically significant.

To determine the level of proinflammatory cytokines, interleukins, and chemokines in the plasma, the blood was collected as soon as the patients were admitted into the hospital and the cytokines were detected with the Luminex assay kit. The relative change rate of main cytokines in JE patients was calculated based on the healthy control (**Figure 2**). The level of IL-18 and IL-6 was significantly elevated in JE patients compared with healthy control which reflected the inflammation and was consistent with previous reports in CNS. Surprisingly, the levels of TNF-α, TRAIL, IL-16, IL-17, and MCP-1 decreased significantly in JE patients which were reported to increase in the brain. The macrophage migration inhibitory factor (MIF) and the monokine induced by interferon-γ (MIG) were also decreased to some extent. The chemokines such as cutaneous T cell-attracting chemokine (CTACK), eotaxin, growth-related oncogene-α (GRO-a), and stromal-derived factor-1α (SDF-1a) were all decreased in JE patients. Meanwhile, the level of stem cell factor (SCF) and platelet-derived growth factor-BB (PDGF-BB) in health individuals was higher than that in JE sufferers.

### The Difference of Main Cytokines in Plasma Among Healthy, Mild, andSevere Patients

**Figure 3:**
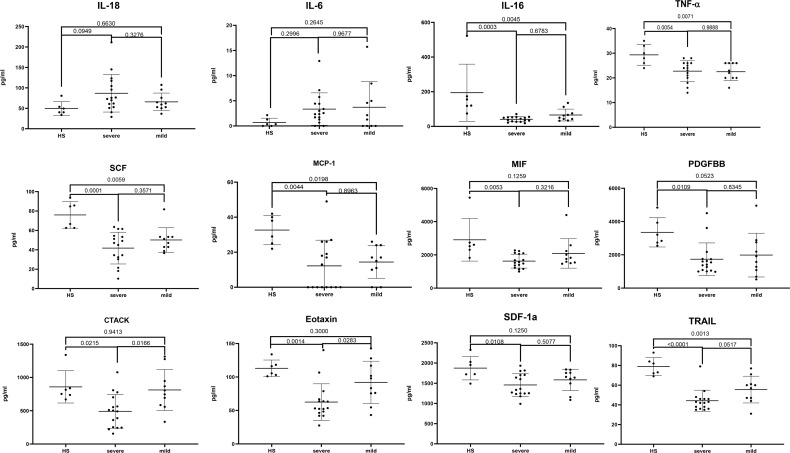
The difference of main cytokines in plasma among healthy, mild, and severe patients. Series of cytokines in the plasma of healthy controls (n = 6); severe JE patients (n = 16, these data were from the plasma of JE cases on the first day they were hospitalized) and mild patients (n = 10) were analyzed with ordinary one-way ANOVA Tukey’s multiple-comparison test. p <0.05 was considered statistically significant.

To clarify the relationship between cytokines and JE severity, the difference of main cytokines in plasma among healthy, mild, and severe patients was analyzed (**Figure 3**). The proinflammatory cytokines IL-18 and IL-6 were relatively higher in JE patients than in the healthy control, while there was no statistic difference between the mild and severe JE patients. Thus, IL-18 and IL-6 were the markers of inflammation but did not reflect the severity of JE. The level of CTACK and eotaxin was decreased significantly in severe JE patients compared with control and mild patients, while there was no significant difference between healthy people and mild JE patients. The level of monocyte chemotactic protein-1 (MCP-1), IL-16, TNF-α, PDGF-BB, SCF, SDF-1a, and vascular endothelial-derived growth factor (VEGF) was much lower in JE mild or severe patients than in healthy people and there was no significant difference between mild and severe sufferers. The level of MIF was greatly decreased compared with healthy people in severe JE individuals but not in mild sufferers. The level of TNF-related apoptosis-inducing ligand (TRAIL) was decreased greatly in both mild and severe JE patients compared to healthy control and was lower in severe individuals than in mild patients.

### Dynamic Changes of Some Main Cytokines in Severe Survived Patients or Fatal Cases

**Figure 4:**
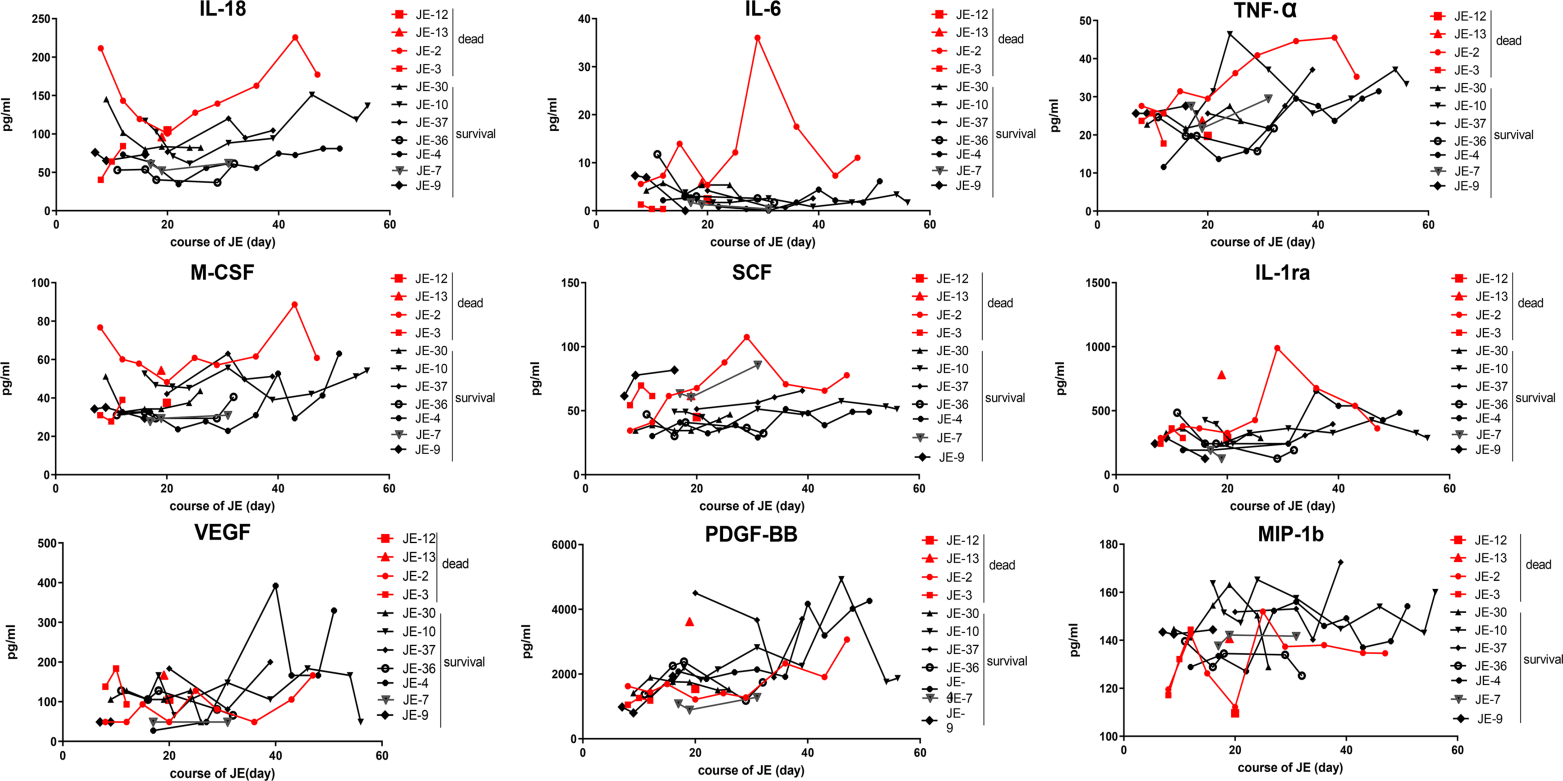
Dynamic changes of some main cytokines in severe survived patients or not. Series of cytokines in the plasma from severe patients without definite nosocomial bacterial infection (four survived cases, seven fatal cases) were monitored continually, and the course of JE was calculated from the onset day of symptoms such as fever and headache according to the complaints.

All the mild JE patients recovered after a few days of hospitalization, while the progression of severe patients was complicated among which some patients survived after a series of treatment and the others died. Thus, we further detected the level of main cytokines dynamically among severe JE patients (**Figure 4**). Four severe patients who failed to survive and seven survivors without definite nosocomial bacterial infection were recruited. Three of the fatal cases showed a pretty high level of IL-18 than did the surviving cases. Two of the fatal cases showed a higher level of IL-6 than did the surviving cases, while there was no significant difference in the classical proinflammatory cytokine TNF-α between the fatal and surviving cases. More than two of the fatal cases also showed higher levels of M-CSF, SCF, and IL-1ra than did the survivors, while the levels of VEGF, MIP-1b, and PDGF-BB were insistently low in dead cases compared to the surviving individuals. There was no significant difference in the levels of MIF, MIG, RANTES, MIP-1a, SDF-1a, and SCGF-β TRAIL between the fatal and survival individuals (**Supplementary Figure 1**).

### Dynamic Changes of the Main Classification of Blood Cells According to the Blood Routine Test in Severe JE Patients Recovery or Not

**Figure 5:**
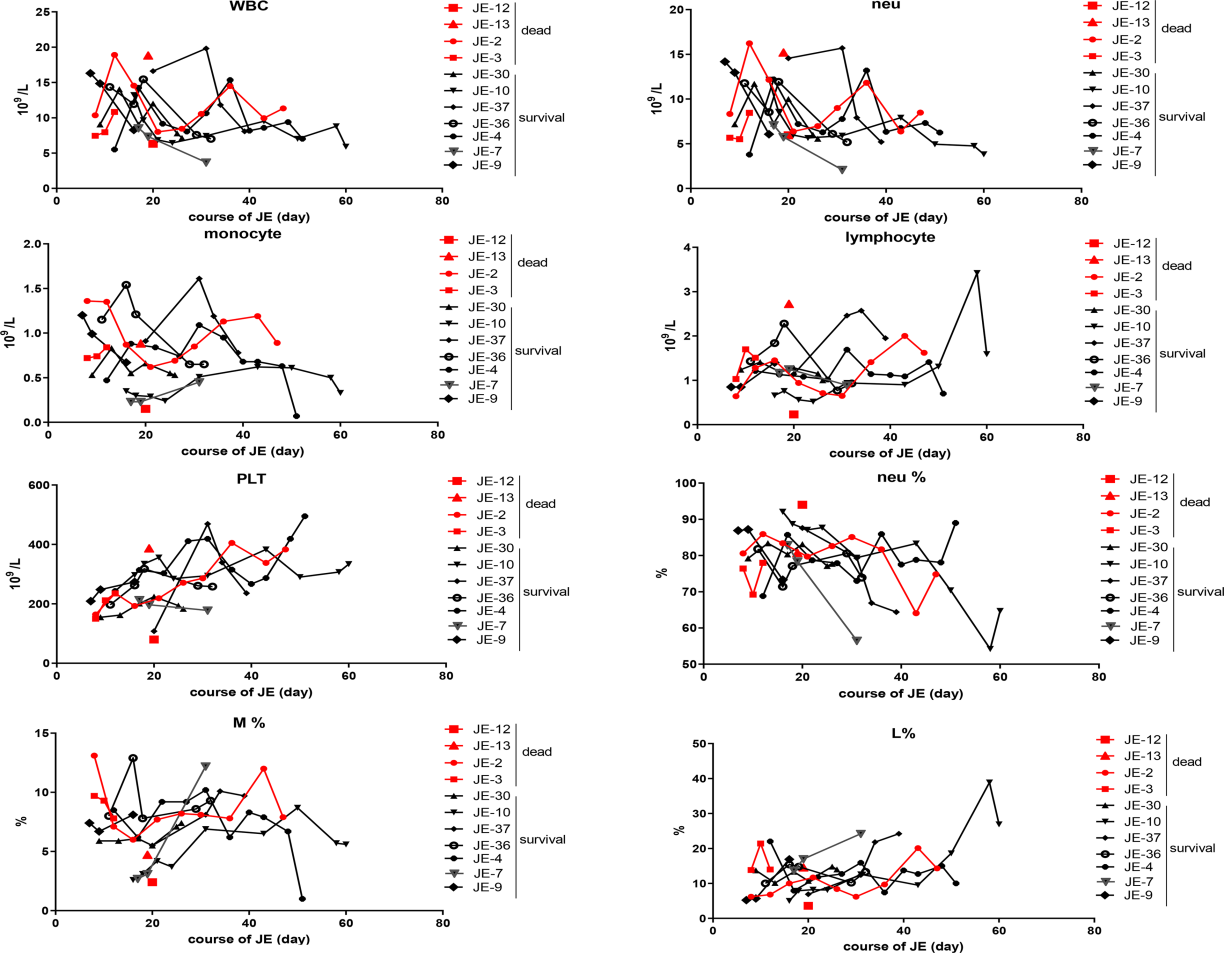
Dynamic changes of the main classification of immune cells according to the blood routine test in severe survived patients or not. The total numbers of white blood cell (WBC), neutrophil (neu), monocyte, lymphocyte, and blood platelet (PLT) from the fatal and survived individuals were counted respectively according to the blood routine test. The percentage of neutrophil, monocyte, and lymphocyte in each case was described continually.

The difference in the blood cells between the fatal and surviving cases was explored (**Figure 5**). The dynamic changes in the main classification of blood cells including WBC, neutrophils, monocytes, lymphocytes, and platelets were analyzed roughly according to the blood routine test, while there was no significant difference in the total WBC, neutrophils, lymphocytes, and monocytes as well as the proportion of the subclass between fatal and surviving individuals.

### The Change in Total T Cells and the Subset in JE Patients With Different Progression

**Figure 6:**
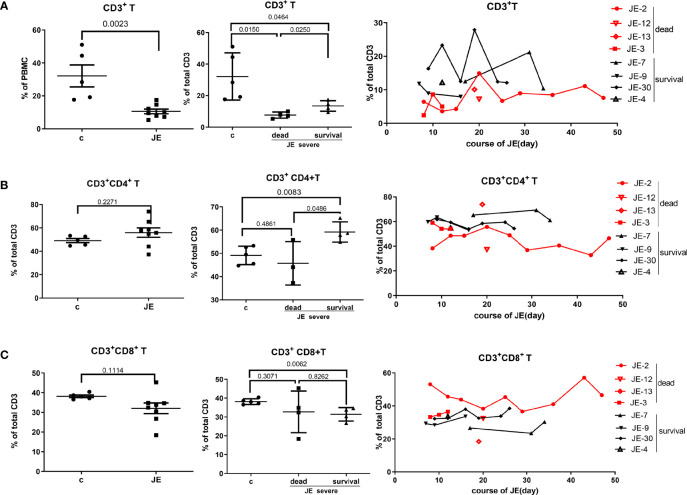
The divergence of T cells and main subsets in JE patients with different progression. The PBMCs from severe JE patients with different outcomes were collected at several time points. The percentages of T cells, CD4^+^ T cells, and CD8^+^ T cells were detected by flow cytometry and analyzed using FlowJo Software v7.6. The data in the severe patients were the mean percentage from several time points of each patient. **(A)** The percentage and comparison of CD3^+^ T cells in the PBMCs from the healthy controls and severe JE patients and the dynamic development of CD3^+^ T cells in the severe JE patients with dead or survival outcome. **(B)** The percentage and comparison of CD3^+^CD4^+^ T cells in the PBMCs from the healthy controls and severe JE patients and the dynamic development of CD3^+^ CD4^+^T cells in the severe JE patients with dead or survival outcome. **(C)** The percentage and comparison of CD3^+^CD8^+^ T cells in the PBMCs from the healthy controls and severe JE patients and the dynamic development of CD3^+^ CD8^+^T cells in the severe JE patients with dead or survival outcome.

To explore the change of T cells in severe JE patients and the difference of T cells between the fatal and survival individuals, the total T cells as well as the subset were detected through flow cytometry. In the severe JE patients, the proportion of total CD3^+^ T cells was decreased (**Figure 6A**) and remained at a lower level in the fatal group. Then, the proportion of the main subset CD4^+^ T cells and CD8^+^ T cells in the total T cells was analyzed further. Generally, there was a comparable proportion of CD4^+^ T cells (**Figure 6B**) as well as CD8^+^ T cells (**Figure 6C**) between the healthy control and severe JE patients, while, among these severe JE patients, the surviving group showed an increased percentage of CD4^+^ T cells (**Figure 6B**) and decreased CD8^+^ T cells (**Figure 6C**) compared with the healthy group. Moreover, there was a higher proportion of CD4^+^ T cells in the surviving individuals than in the fatal group (**Figure 6B**). Meanwhile, dynamic variations of CD4^+^ T cells and CD8^+^ T cells were detected. The surviving JE patients showed a continued higher percentage of CD4^+^ T cells (**Figure 6B**) and a lower proportion of CD8^+^ T cells (**Figure 6C**) than the fatal individuals. In summary, the proportion of CD3^+^ T cells was lower in the severe JE patients than in the healthy group. Moreover, the percentage of CD4^+^ T cells was increased and the proportion of CD8^+^ T cells was decreased in the surviving individuals compared with the fatal group.

### B Cells, Monocytes, and NK Cells Decreased Significantly in JE Patients With Fatal Progression

**Figure 7:**
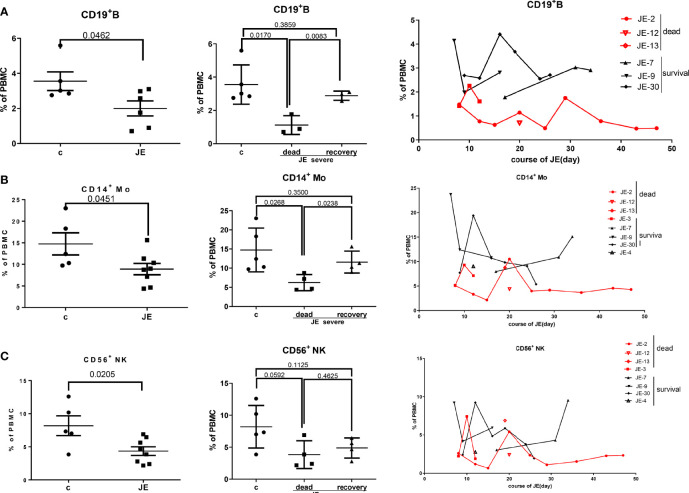
The number of B cells, monocytes, and NK cells decreased significantly in JE patients with fatal progression. The PBMCs from severe JE patients with different outcomes were collected at several time points. The percentages of CD19^+^ B cells, CD14^+^ monocytes, and CD56^+^ NK cells were detected by flow cytometry and analyzed using FlowJo Software v7.6. The data in the severe patients were the mean percentage from several time points of each patient. **(A)** The percentage, comparison, and dynamic development of CD19^+^ B cells in the PBMCs from the healthy controls and severe JE patients. **(B)** The percentage, comparison and dynamic development of CD14^+^ monocytes in the PBMCs from the healthy controls and severe JE patients. **(C)** The percentage, comparison, and dynamic development of CD56^+^ NK cells in the PBMCs from the healthy controls and severe JE patients.

It has been reported that CD4^+^ T cells promoted humoral immunity and viral control (Turtle et al., 2016; Elong Ngono et al., 2019). Then, we detected the change of humoral immunity in the progression of severe JE. The proportion of CD19^+^ B cells in the PBMC from fatal and surviving JE patients was detected through flow cytometry. Compared to the healthy control, the proportion of B cells was decreased in the severe JE patients especially in the fatal cases (**Figure 7A**). Meanwhile, the fatal cases showed a much lower proportion of B cells than the surviving cases. Further, the dynamic change of B cells in the severe fatal and surviving JE patients was analyzed. A continuously low proportion of B cells was found in the fatal group. In summary, B cells decreased significantly in severe JE patients with fatal progression.

The peripheral immune system is activated and contributes to the elimination of virus during early infection. To explore the variance of peripheral innate immunity in the severe JE patients with different progression, the proportion of monocytes and NK cells in the PBMC was detected. The percentages of both CD14^+^ monocytes (**Figure 7B**) and the CD56^+^ NK cells (**Figure 7C**) were decreased in the severe JE patients. Moreover, the proportion of CD14^+^ monocytes in the fatal JE group was significantly lower than in the healthy group (**Figure 7B**) and a maintained low percentage compared with the surviving JE individuals, while there was no statistic difference in the proportion of NK cells between the surviving and fatal severe JE patients (**Figure 7C**).

## Discussion

Although the incidence of JE was reduced significantly since the launch of the JEV vaccination program, small-scale dissemination of infection still occurred irregularly especially in the adults. The absence of effective and specific therapy for JE patients resulted in about 25%–30% of JE fatal cases and 50% permanent neuropsychiatric sequelae (Campbell et al., 2011). To better understand the correlation of JE clinical progression and peripheral inflammation, the main cytokines and immune cells in the blood from healthy control and JE patients with different progression were detected and analyzed. There were no correlations between age and the severity of JE-positive patients. The levels of IL-18 and IL-6 in the JE patients were increased significantly especially in the fatal cases. Compared with the surviving individuals, the fatal cases showed an insistently lower proportion of CD4^+^ T cells and B cells. According to these results, we speculated that consistently high levels of IL-6 and IL-18 and a low proportion of CD4^+^ T and B cells in blood of JE patients indicated poor prognosis.

Neuroinflammation is the main characteristic of JE. Compared with healthy controls, IL-6 and IL-18 in the plasm were increased in the JE patients, while the proinflammatory cytokines TNF-α and IL-17 were lower in the JE patients as well as a series of chemokines such as MCP-1, CTACK, eotaxin, and GRO-a and growth factors PDGF-BB, SCF, and SDF-1a. On the one hand, there were discrepancies in the cytokines between the plasma and cerebrospinal fluid which reflected the neuroinflammation better. On the other hand, in this study, the data were from the mean of different stages in the JE progression instead of the exact acute phase. Interestingly, the chemokines CTACK and eotaxin were decreased significantly in the plasma of severe JE patients compared with healthy controls and mild patients which needed to be explored further.

In order to identify the key cytokines related to the JE prognosis, severe JE patients without secondary infection were recruited and the cytokines in the plasm of surviving and fatal individuals were analyzed retrospectively. Most fatal individuals showed higher levels of IL-18, IL-6, TNF-α, M-CSF, SCF, and IL-1ra continually than the surviving cases in the same phase, while VEGF, PDGF-BB, and MIP-1b maintained relatively lower levels in the fatal cases. These cytokines might be the candidates as the predictors of severe JE progression, and more cases should be recruited for further verification.

IL-6 was increased in the JE patients, and most fatal individuals showed a higher level of IL-6. While several studies show the essential role of IL-6 to mount a proper immune response during some viral infections, others link this cytokine with exacerbation of viral diseases (Velazquez-Salinas et al., 2019). Our data support the hypothesis that upregulation of IL-6 during JEV infection may exacerbate the disease, and IL-6 may suppress CD8 T-cell immunity.

Immune cells play important roles in the eradication of virus and JE recovery. Observation from mouse models indicated that CD4^+^ T cells played an important role in the progression of JE but not CD8^+^ T cells (Wang et al., 2017). Meanwhile, a series of retrospective studies in convalescent JE patients demonstrated that the completely recovered patients showed a higher proportion of IFN-γ-dominated CD4^+^ T cells than the poor-outcome cases (Turtle et al., 2016), while the dynamic change of the T cells and JE progression in severe cases was unclear. In this study, the percentages of CD4^+^ and CD8^+^ T cells in the PBMCs of severe JE patients with different prognoses were monitored continually. Consistent with previous animal studies (Larena et al., 2011; Wang et al., 2017), the surviving JE patients showed a higher percentage of CD4^+^ T cells but not CD8^+^ T cells than the fatal cases. What is more, the suppression of total T-cell immunity was found in severe JE patients especially in the fatal groups, while, compared with the healthy control and fatal groups, most of the surviving individuals showed increased CD4^+^ T-cell immunity and decreased CD8^+^ T-cell proportion.

It has been reported that CD4^+^ T cells promote humoral immunity and viral control during Zika virus infection (Elong Ngono et al., 2019). Meanwhile, it has been demonstrated in the JEV-infected mouse models that JEV infection-induced myeloid-derived suppressor cells (MDSCs) could suppress CD4^+^ T-cell immune responses then lead to decreased B-cell populations and humoral immunity and finally facilitate the progression of infection (Wang et al., 2017). In this study, we found that the percentages of CD4^+^ T cells and B cells in the PBMCs were decreased in the severe JE patients especially the fatal cases. Thus, we speculated that a lower percentage of CD4^+^ T cells in the early infection might contribute to decreased B-cell proliferation and eventually the fatal prognosis in JE patients. Continuous population monitoring of CD4^+^ T cells and B cells shows that they might be the indicators of severe JE prognosis.

It has been demonstrated that innate immune NK cells and monocytes played complicated roles in the JEV control as well as immunomodulation at the periphery and CNS. It was reported that JEV modulates suppressors of cytokine signaling (SOCS) 1 and 3 expression in macrophages to bring about changes in the JAK-STAT signaling cascade, so as to inhibit proinflammatory cyto/chemokine release (Kundu et al., 2013). IL12p70, TNF-α, and IL-6 release by peritoneal macrophages post-JEV infection was significantly high. In our study, compared with healthy controls, the severe JE patients showed a reduced percentage of NK cells and monocytes in the PBMCs. Meanwhile, the innate immune cells in most of the surviving individuals were higher than in the fatal cases during the severe JE progression.

Immune responses of the vaccinated population may differ from those of the naïve population upon natural infection. A live attenuated JE vaccine (SA-14-14-2) was successfully developed in 1989 in China; free and compulsory JE vaccines were used for children aged 8 months and 2 years. We could estimate that most patients over 30 years were not vaccinated. Most severe patients admitted into the ICU were over 30 years old except one who was aged 20. The ages of fatal patients were 52, 53, 65, and 67 while the ages of surviving patients were 20, 36, 52, 54, 57, 60, and 71. There was no statistical significance of age between fetal and surviving JE patients. If the fatal cases were naïve and the surviving severe cases were immunized, the interpretation of the data might be affected, which needs further study.

There are five genotypes of JEV; genotype-l is the main prevailing genotype. Since the JEV is mainly found in brain tissue, with very low titer of virus in blood or cerebrospinal fluid, we did not isolate the virus to analyze the genotype. The genotype of JEV might affect the outcome of the patient after infection. More experiments are worth performing.

Conclusively, in this cohort study of JE patients, we found that the fatal JE cases showed consistently high levels of IL-6 and IL-18 in the plasm and low proportions of CD4^+^ T and B cells in the PBMCs which might be the indicators of poor prognosis. Meanwhile, the exact correlation of innate immune cells and adaptive immune cells as well as the severe JE prognosis needs to be explored further.

## Data Availability Statement

The original contributions presented in the study are included in the article/**Supplementary Material**. Further inquiries can be directed to the corresponding authors.

## Ethics Statement

The research protocols were approved by the Scientific and Ethical Committee of Tangdu Hospital. The patients/participants provided their written informed consent to participate in this study.

## Author Contributions

YZ and PB performed most of the experiments. HD, TW, and ML helped collect clinical data. HH, CY, and XZ helped analyze the data. YZ and YL provided technical support and offered intellectual input for troubleshooting; ZJ and JL supervised the project and wrote the manuscript, with the help of all other authors. All authors contributed to the article and approved the submitted version.

## Funding

This work was supported by the Major National Science and Technology Projects (2017ZX10204401-002-005).

## Conflict of Interest

The authors declare that the research was conducted in the absence of any commercial or financial relationships that could be construed as a potential conflict of interest.

## Publisher’s Note

All claims expressed in this article are solely those of the authors and do not necessarily represent those of their affiliated organizations, or those of the publisher, the editors and the reviewers. Any product that may be evaluated in this article, or claim that may be made by its manufacturer, is not guaranteed or endorsed by the publisher.
