# Supplementary material for: The Comparison of Inflammatory Cytokines (IL-6 and IL-18) and Immune Cells in Japanese Encephalitis Patients With Different Progression
Source: Front Cell Infect Microbiol. 2022 Apr 7;12:826603. doi: 10.3389/fcimb.2022.826603 (PMC9022626; doi:10.3389/fcimb.2022.826603)
Supplement: Supplementary file 1 [file DataSheet_1.docx]

Supplementary Material

**
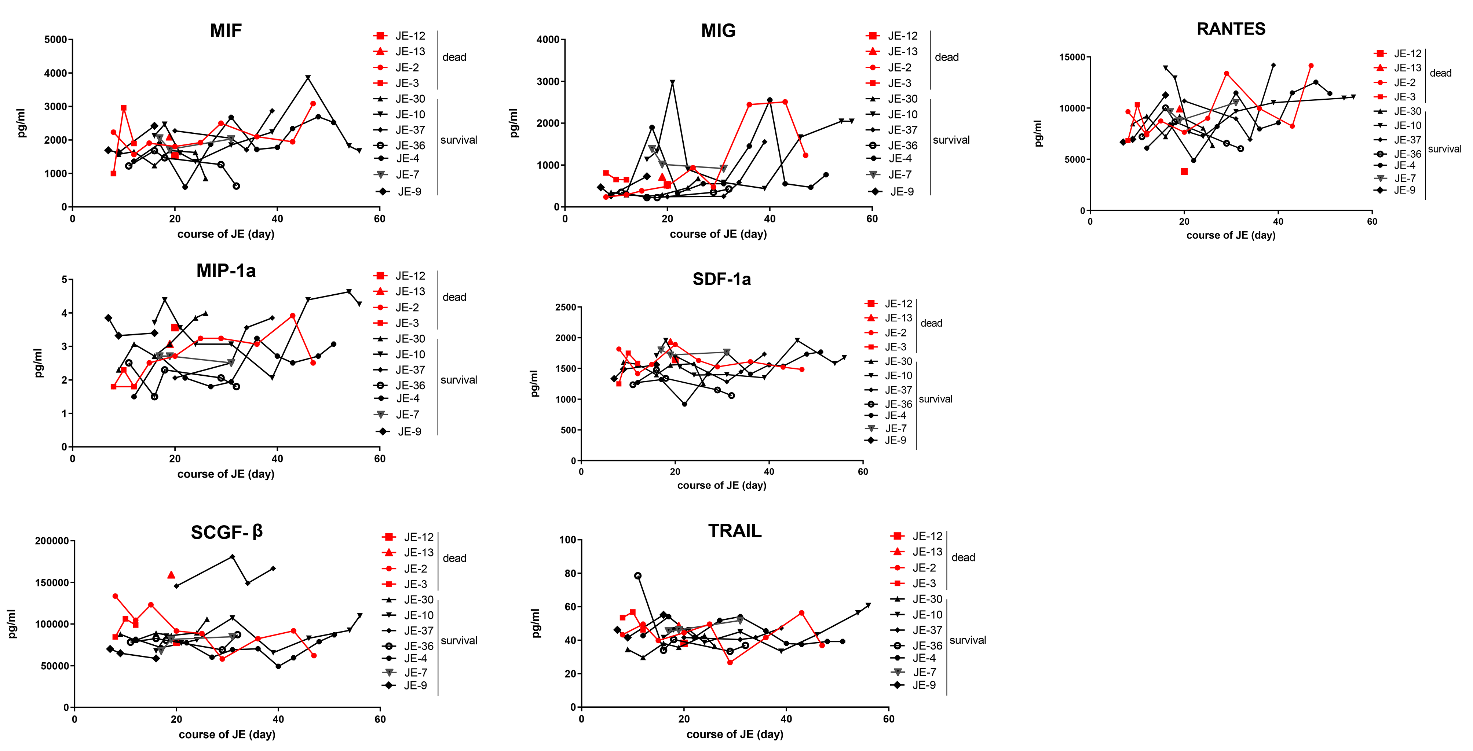
**

**Supplementary Figure 1.** Difference in the level of MIF, MIG, RANTES, MIP-1a, SDF-1a, SCGF-β TRAIL between the fatal and survival individuals
