# Supplementary figures and images for: The Comparison of Inflammatory Cytokines (IL-6 and IL-18) and Immune Cells in Japanese Encephalitis Patients With Different Progression
Source: Front Cell Infect Microbiol. 2022 Apr 7;12:826603. doi: 10.3389/fcimb.2022.826603 (PMC9022626; doi:10.3389/fcimb.2022.826603)

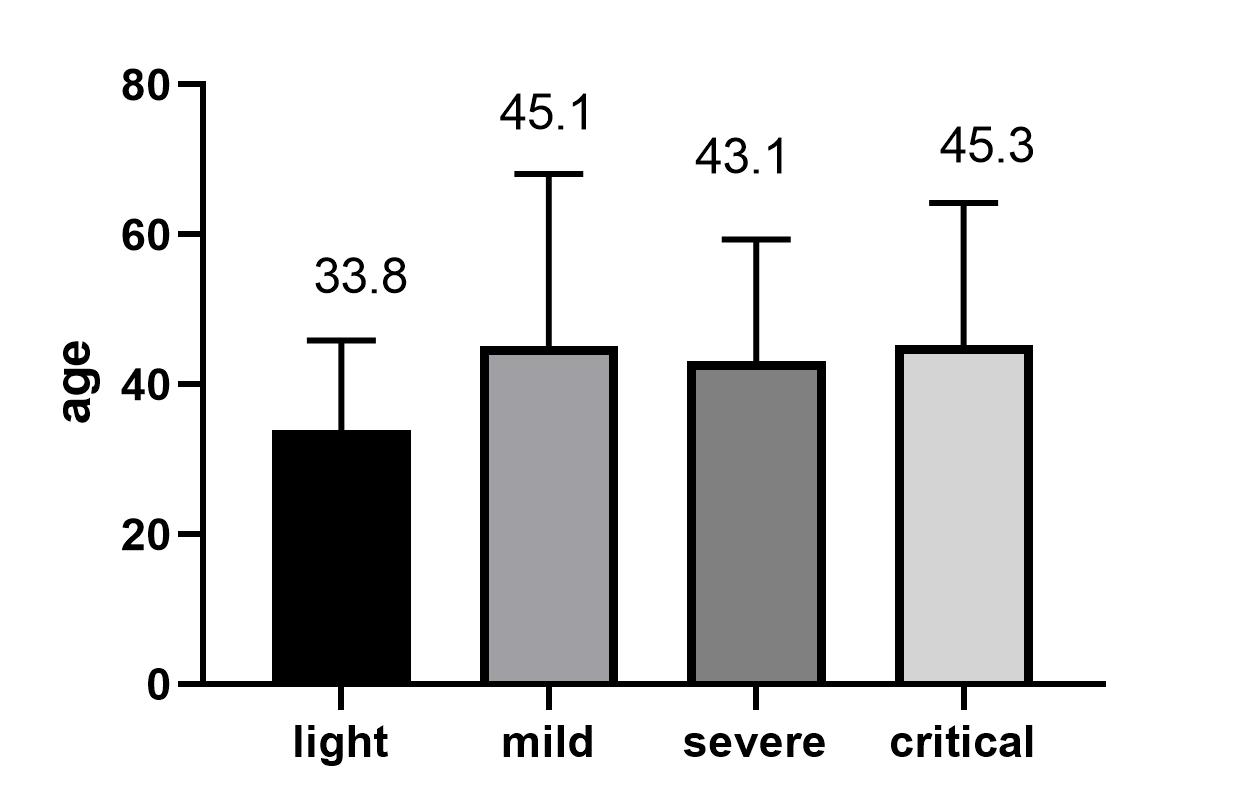

Supplement: Supplementary file 2 [file Image_1.jpeg]
